# Machine Learning and Artificial Intelligence: Two Fellow Travelers on the Quest for Intelligent Behavior in Machines

**DOI:** 10.3389/fdata.2018.00006

**Published:** 2018-11-19

**Authors:** Kristian Kersting

**Affiliations:** Machine Learning Lab, CS Department and Centre for Cognitive Science, Darmstadt University of Technology, Darmstadt, Germany

**Keywords:** machine learning, artificial intelligence, deep learning, computation, learning methods

## 1. Big data is boosting intelligent behavior in machines

Machine learning (ML) and artificial intelligence (AI) are becoming dominant problem-solving techniques in many areas of research and industry, not least because of the recent successes of deep learning (DL). However, the equation AI=ML=DL, as recently suggested in the news, blogs, and media, falls too short. These fields share the same fundamental hypotheses: computation is a useful way to model intelligent behavior in machines. What kind of computation and how to program it? This is not the right question. Computation neither rules out search, logical, and probabilistic techniques, nor (deep) (un)supervised and reinforcement learning methods, among others, as computational models do include all of them. They complement each other, and the next breakthrough lies not only in pushing each of them but also in combining them.

Big Data is no fad. The world is growing at an exponential rate and so is the size of the data collected across the globe. Data is becoming more meaningful and contextually relevant, breaking new grounds for machine learning (ML), in particular for deep learning (DL) and artificial intelligence (AI), moving them out of research labs into production (Jordan and Mitchell, 2015). The problem has shifted from collecting massive amounts of data to understanding it—turning it into knowledge, conclusions, and actions. Multiple research disciplines, from cognitive sciences to biology, finance, physics, and social sciences, as well as many companies believe that data-driven and “intelligent” solutions are necessary to solve many of their key problems. High-throughput genomic and proteomic experiments can be used to enable personalized medicine. Large data sets of search queries can be used to improve information retrieval. Historical climate data can be used to understand global warming and to better predict weather. Large amounts of sensor readings and hyperspectral images of plants can be used to identify drought conditions and to gain insights into when and how stress impacts plant growth and development and in turn how to counterattack the problem of world hunger. Game data can turn pixels into actions within video games, while observational data can help enable robots to understand complex and unstructured environments and to learn manipulation skills.

However, is AI, ML, and DL really synonymous, as recently suggested in the news, blogs, and media? For example, when AlphaGo (Silver et al., 2016) defeated South Korean Master Lee Se-dol in the board game Go in 2016, the terms AI, ML, and DL were used by the media to describe how AlphaGo won. In addition to this, even Gartner's list (Panetta, 2017) of top 10 Strategic Trends for 2018 places (narrow) AI at the very top, specifying it as “consisting of highly scoped machine-learning solutions that target a specific task.”

## 2. Artificial intelligence and machine learning

Artificial intelligence and ML are very much related. According to McCarthy (2007), one of the founders of the field,

AI is “the science and engineering of making intelligent machines, especially intelligent computer programs. It is related to the similar task of using computers to understand human intelligence, but AI does not have to confine itself to methods that are biologically observable.”

This is fairly generic and includes multiple tasks such as abstractly reasoning and generalizing about the world, solving puzzles, planning how to achieve goals, moving around in the world, recognizing objects and sounds, speaking, translating, performing social or business transactions, creative work (e.g., creating art or poetry), and controlling robots. Moreover, the behavior of a machine is not just the outcome of the program, it is also affected by its “body” and the enviroment it is physically embedded in. To keep it simple, however, if you can write a very clever program that has, say, human-like behavior, it can be AI. But unless it automatically learns from data, it is not ML:

ML is the science that is “concerned with the question of how to construct computer programs that automatically improve with experience,” (Mitchell, 1997).

So, AI and ML are both about constructing intelligent computer programs, and DL, being an instance of ML, is no exception. Deep learning (LeCun et al., 2015; Goodfellow et al., 2016), which has achieved remarkable gains in many domains spanning from object recognition, speech recognition, and control, can be viewed as constructing computer programs, namely programming layers of abstraction in a differentiable way using reusable structures such as convolution, pooling, auto encoders, variational inference networks, and so on. In other words, we replace the complexity of writing algorithms, that cover every eventuality, with the complexity of finding the right general outline of the algorithms—in the form of, for example, a deep neural network—and processing data. By virtue of the generality of neural networks—they are general function approximators—training them is data hungry and typically requires large labeled training sets. While benchmark training sets for object recognition, store hundreds or thousands of examples per class label, for many AI applications, creating labeled training data is the most time-consuming and expensive part of DL. Learning to play video games may require hundreds of hours of training experience and/or very expensive computing power. In contrast, writing an AI algorithm that covers every eventuality of a task to solve, say, reasoning about data and knowledge to label data automatically (Ratner et al., 2016; Roth, 2017) and, in turn, make, for example, DL less data-hungry–is a lot of manual work, but we know what the algorithm does by design and that it can study and that it can more easily understand the complexity of the problem it solves. When a machine has to interact with a human, this seems to be especially valuable.

This illustrates that ML and AI are indeed similar, but not quite the same. Artificial intelligence is about problem solving, reasoning, and learning in general. Machine learning is specifically about learning—learning from examples, from definitions, from being told, and from behavior. The easiest way to think of their relationship is to visualize them as concentric circles with AI first and ML sitting inside (with DL fitting inside both), since ML also requires writing algorithms that cover every eventuality, namely, of the learning process. The crucial point is that they share the idea of using computation as the language for intelligent behavior. What kind of computation is used and how should it be programed? This is not the right question. Computation neither rules out search, logical, probabilistic, and constraint programming techniques nor (deep) (un)supervised and reinforcement learning methods, among others, but does, as a computational model, contain all of these techniques.

Reconsidering AlphaGo: AlphaGo and its successor AlphaGo Zero (Silver et al., 2017) both combine DL and tree search—ML and AI. Alternatively, the “Allen AI Science Challenge” (Schoenick et al., 2017) should be considered. The task was to comprehend a paragraph that states a science problem, at the middle school level and then to answer a multiple-choice question. All winning models employed ML yet failed to pass the test at the level of a competent middle schooler. All winners argued that it was clear that applying a deeper, semantic level of reasoning with scientific knowledge to the question and answers, is the key to achieving true intelligence. In other words, AI has to cover knowledge, reasoning, and learning, using programmed and learning-based programmed models in a combined fashion.

## 3. The joint quest to identify intelligent behavior in machines

Using computation as the common language, we have come a long way, but the journey ahead is still long. None of today's intelligent machines come close to the breadth and depth of human intelligence. In many real-world applications, as illustrated by AlphaGo and the Allen AI Science Challenge, it is unclear whether problem formulation falls neatly into fully learning. The problem may well have a large component, which can be best modeled using an AI algorithm without the learning component, but there may be additional constraints or missing knowledge that take the problem outside its regime, and learning may help to fill the gap. Similarly, programmed knowledge and reasoning may help learners to fill their gaps. There is a symmetric difference between AI and ML, and intelligent behavior in machines is a joint quest, with many vast and fascinating open research problems:

- How can computers reason about and learn with complex data such as multimodal data, graphs, and uncertain databases?
- How can preexisting knowledge be exploited?
- How can we ensure that learning machines fulfill given constraints and provide certain guarantees?
- How can computers autonomously decide the best representation for the data at hand?
- How do we orchestrate different algorithms, involving learned or not learned ones?
- How do we democratize ML and AI?
- Can learned results be physically plausible or easily understood by us?
- How do we make computers learn with us in the loop?
- How do we make computers learn with less help and data provided by us?
- Can they autonomously decide the best constraints and algorithms for a task at hand?
- How do we make computers learn as much about the world, in a rapid, flexible, and explainable manner, as humans?

Answering these and other similar questions will put the dream of intelligent and responsible machines into reach. Fully programmed computations, together with learning-based programmed computations, will help to better generalize, beyond the specific data that we have seen, whether a new pronunciation of a word or an image will significantly differ from those we have seen before. They allow us to go significantly beyond supervised learning, towards incidential and unsupervised learning, which does not depend so much on labeled training data. They provide a common ground for continuous, deep, and symbolic manipulations. They allow us to derive insights from cognitive science and other disciplines for ML and AI. They allow us to focus more on acquiring common sense knowledge and scientific reasoning, while also providing a clear path for democratizing ML-AI technology, as suggested by De Raedt et al. (2016) and Kordjamshidi et al. (2018). Building intelligent systems requires expertise in computer science and extensive programming skills to work with various machine reasoning and learning techniques at a rather low-level of abstraction. Building intelligent systems also requires extensive trial and error exploration for model selection, data cleaning, feature selection, and parameter tuning. There is actually a lack of theoretical understanding that could be used to remove these subtleties. Conventional programming languages and software engineering paradigms have also not been designed to address the challenges faced by AI and ML practitioners, such as dealing with messy, real-world data at the right level of abstraction and with constantly changing problem definitions. Finally, data-driven science is an exploratory task. Starting from a substantial foundation of domain expert knowledge, relevant concepts as well as heuristic models can change, and even the problem definition is likely to be reshaped concurrently in light of new evidence. Interactive ML and AI can form the basis for new methods that model dynamically evolving targets and incorporate expert knowledge on the fly. To allow the domain expert to steer data-driven research, the prediction process additionally needs to be sufficiently transparent.

## 4. Conclusions

Machine learning and AI complement each other, and the next breakthrough lies not only in pushing each of them but also in combining them. Our algorithms should support (re)trainable, (re)composable models of computation and facilitate reasoning and interaction with respect to these models at the right level of abstraction. Multiple disciplines and research areas need to collaborate to drive these breakthroughs. Using computation as the common language has the potential for progressing learning concepts and inferring information that is both easy and difficult for humans to acquire.

To this end, the “Machine Learning and Artificial intelligence” section in Frontiers in Big Data welcomes foundational and applied papers as well as replication studies from a wide range of topics underpinning ML, AI, and their interplay. It will foster the scholarly discussion of the causes and effects of achievements providing a proper perspective on the obtained results. Using the common language of computation, we can fully understand how to achieve intelligent behavior in machines.

## Author contributions

The author confirms being the sole contributor of this work and has approved it for publication.

### Conflict of interest statement

The author declares that the research was conducted in the absence of any commercial or financial relationships that could be construed as a potential conflict of interest.
